# Supplementary material for: Addressing initialisation uncertainty for end-to-end ecosystem models: application to the Chatham Rise Atlantis model
Source: PeerJ. 2020 Jun 3;8:e9254. doi: 10.7717/peerj.9254 (PMC7292022; doi:10.7717/peerj.9254)
Supplement: Supplemental Information 1 [file peerj-08-9254-s001.pdf]

Biomass trajectories

Biomass trajectories from models with fishing included (blue lines) and no fishing (orange lines) for each species group, with CVs from across the model runs by time from fished models (midnight blue crosses) and unfished models (dark orange pluses) overlaid and using the right-hand axis.

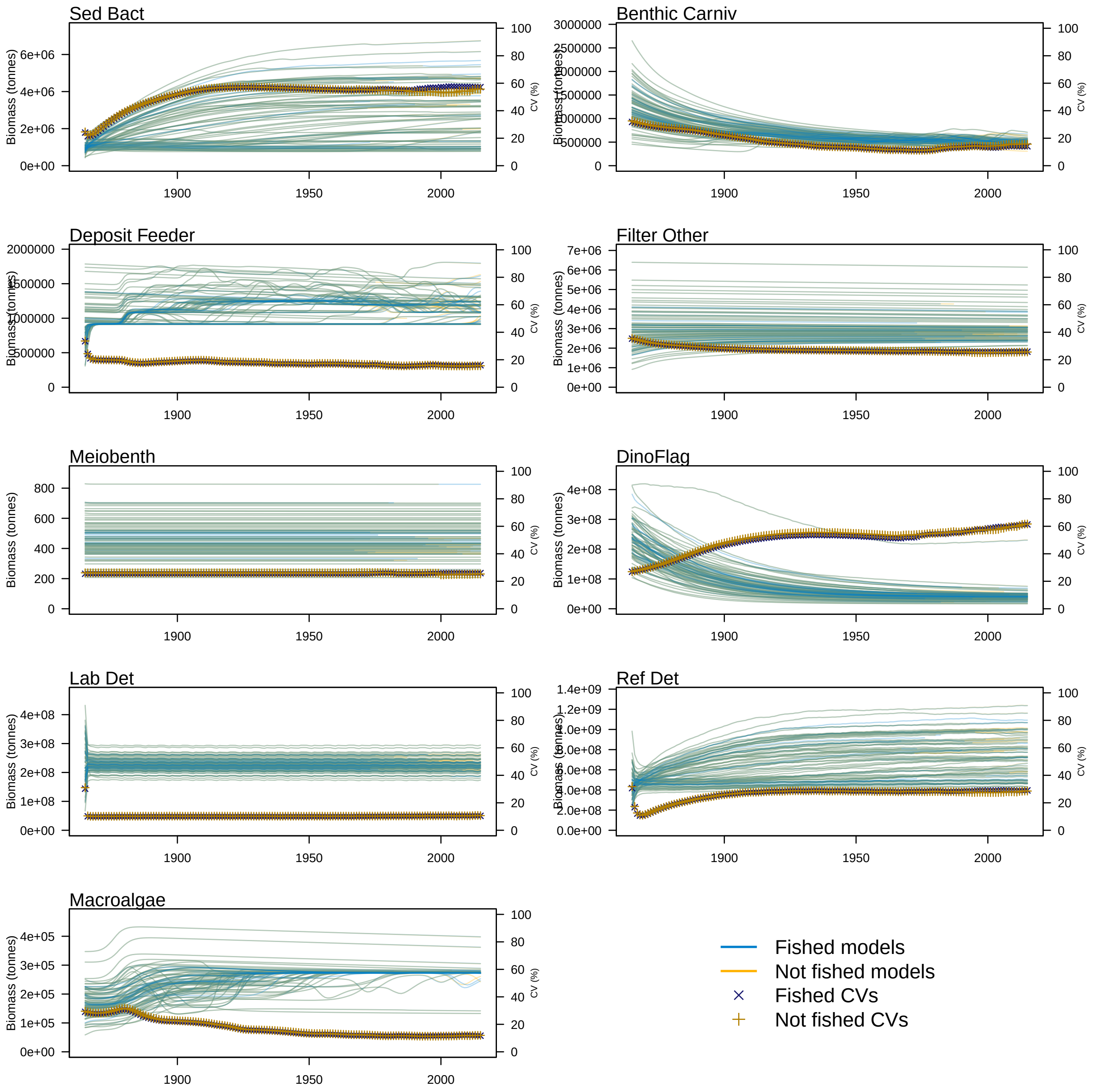

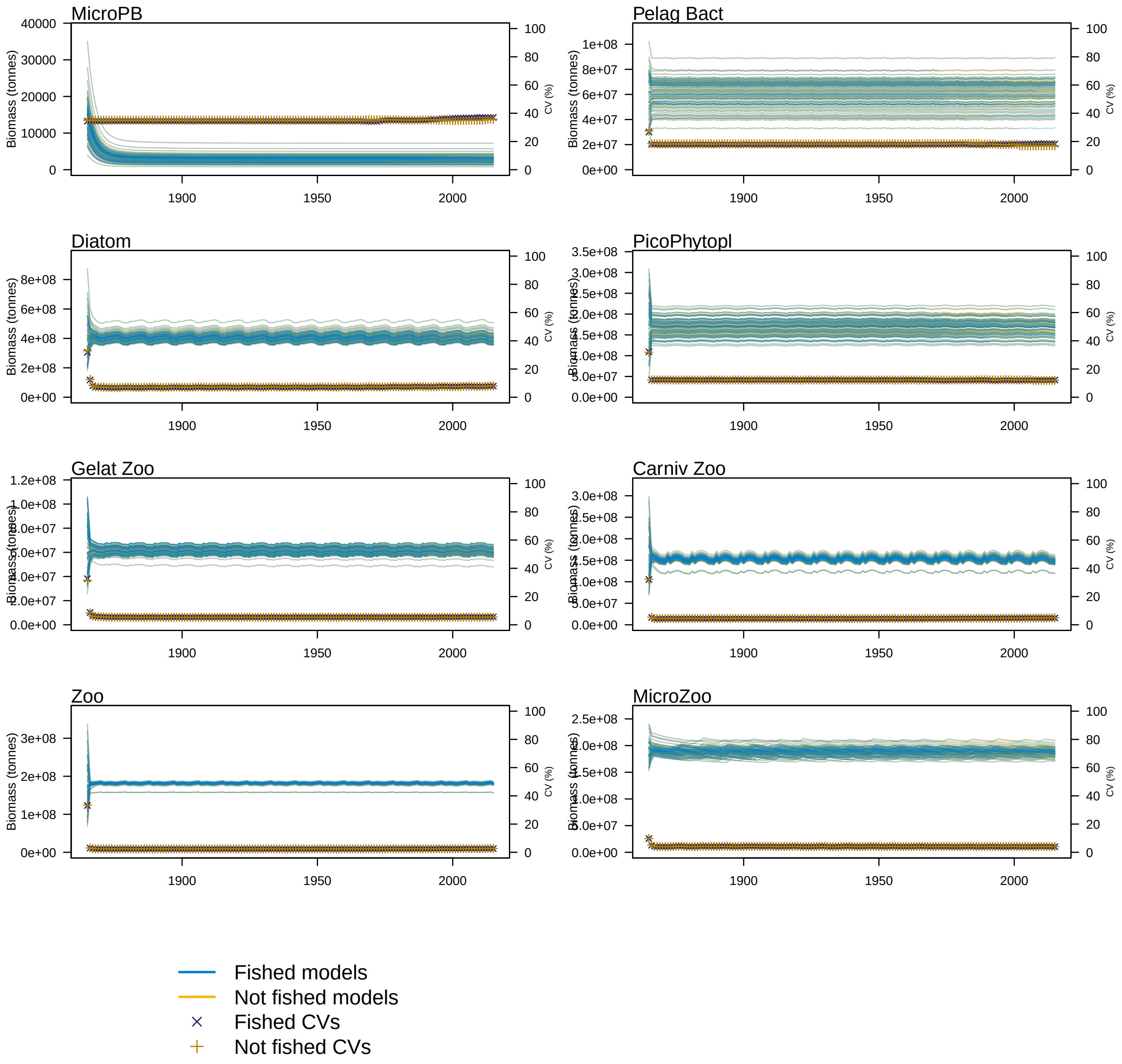
MicroPB
Pelag Bact
Diatom
PicoPhytopl
Gelat Zoo
Carniv Zoo
Zoo
MicroZoo
Biomass (tonnes)
CV (%)
1900
1950
2000
Fished models
Not fished models
Fished CVs
Not fished CVs

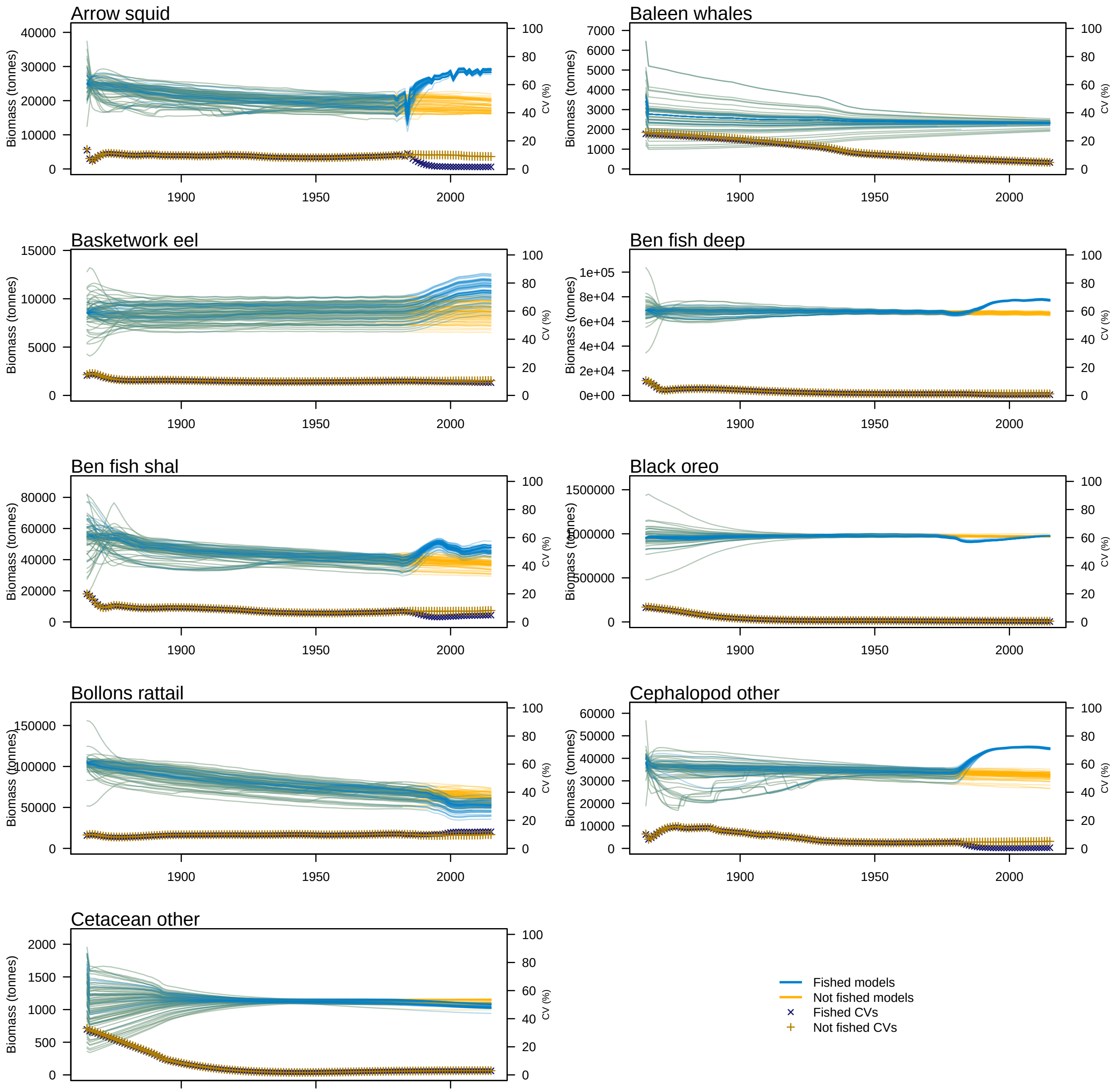

Arrow squid
Baleen whales
Basketwork eel
Ben fish deep
Ben fish shal
Black oreo
Bollons rattail
Cephalopod other
Cetacean other
Biomass (tonnes)
CV (%)
Fished models
Not fished models
Fished CVs
Not fished CVs

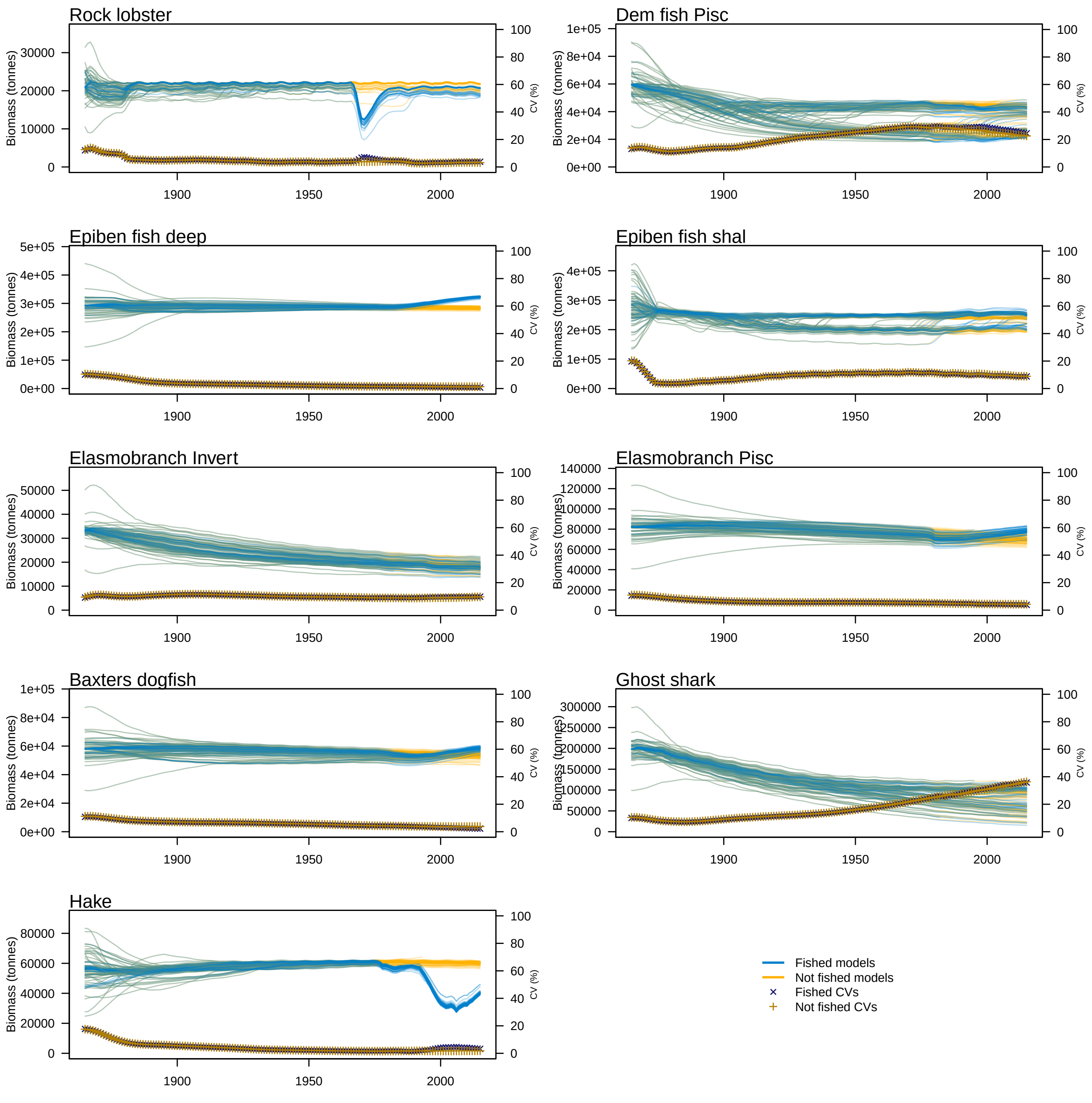
Rock lobster
Dem fish Pisc
Epiben fish deep
Epiben fish shal
Elasmobranch Invert
Elasmobranch Pisc
Baxters dogfish
Ghost shark
Hake
Biomass (tonnes)
CV (%)
1900
1950
2000
Fished models
Not fished models
Fished CVs
Not fished CVs

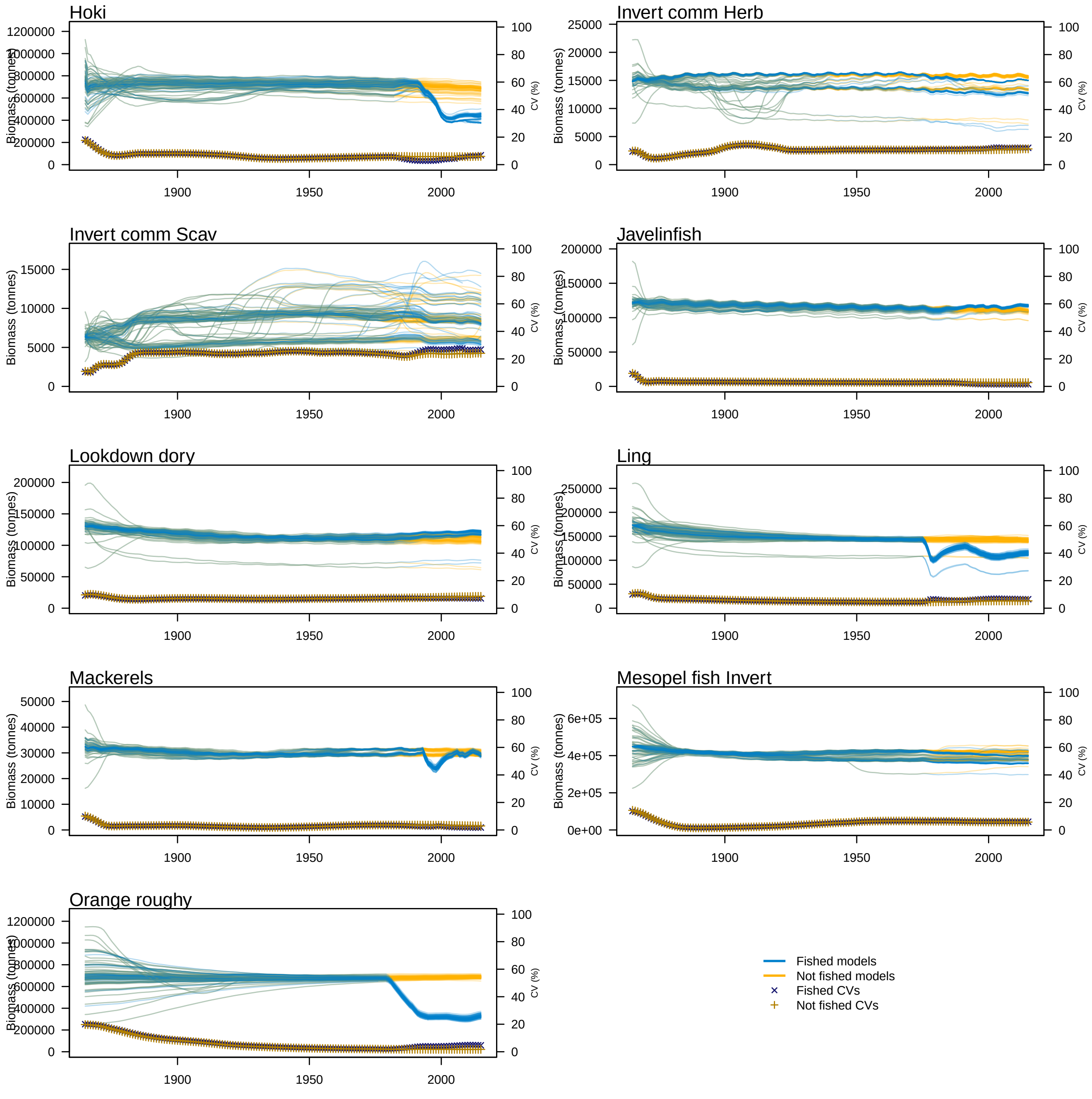
Hoki
Invert comm Herb
Invert comm Scav
Javelinfish
Lookdown dory
Ling
Mackerels
Mesopel fish Invert
Orange roughy
Biomass (tonnes)
CV (%)
1900
1950
2000
Fished models
Not fished models
Fished CVs
Not fished CVs

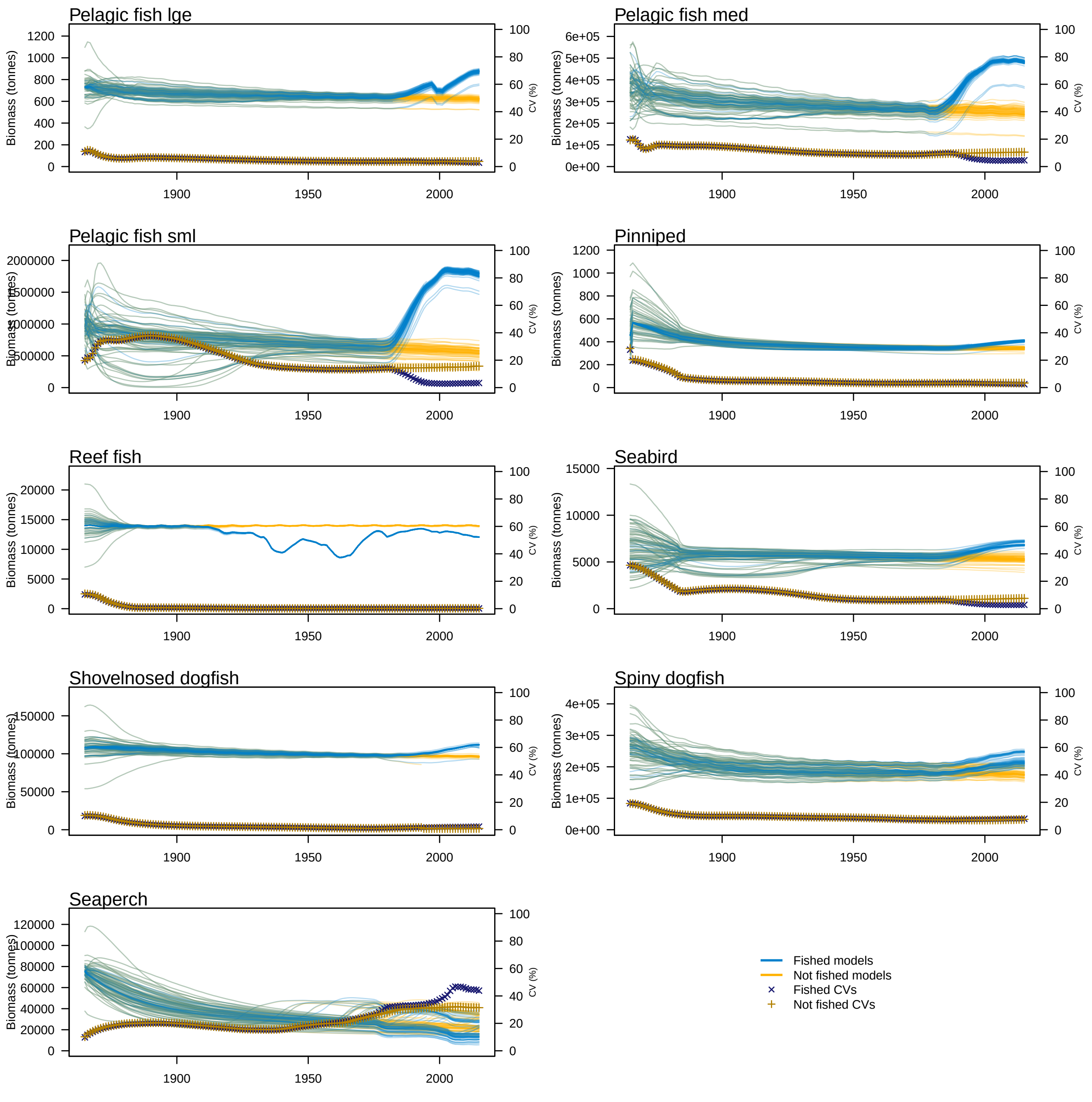
Pelagic fish lge
Pelagic fish med
Pelagic fish sml
Pinniped
Reef fish
Seabird
Shovelnosed dogfish
Spiny dogfish
Seaperch
Biomass (tonnes)
CV (%)
1900
1950
2000
Fished models
Not fished models
Fished CVs
Not fished CVs
